# Book review of "The Ethics of Coercion in Mass Casualty Medicine" by Griffin Trotter MD, PhD

**DOI:** 10.1186/1747-5341-2-20

**Published:** 2007-10-02

**Authors:** Sonal Singh

**Affiliations:** 1 Department of Medicine, Wake Forest University School of Medicine, Winston-Salem, NC 27103, USA

## Abstract

Public health ethics is neither taught widely in medical schools or schools of public health in the US or around the world. It is not surprising that health care professionals are particularly challenged when faced with ethical questions which extend beyond safeguarding the interests of their individual patients to matters that affect overall public good. The perceived threat of terror after September 11 2007, the anthrax attacks and the Katrina debacle are recent circumstances which may result in coercion. These have piqued the interest of medical professionals and the general public on public health ethics. *The Ethics of Coercion in Mass Casualty Medicine *written by Griffin Trotter MD, PhD attempts to fill a timely void in this area by examining the ethics of coercion in times of public health disasters.

## 

Public health ethics is neither taught widely in medical schools or schools of public health in the US or around the world. It is not surprising that health care professionals are particularly challenged when faced with ethical questions which extend beyond safeguarding the interests of their individual patients to matters that affect overall public good. The perceived threat of terror after September 11 2007, the anthrax attacks and the Katrina debacle are recent circumstances, which may result in coercion. These have piqued the interest of medical professionals and the general public on public health ethics. *The Ethics of Coercion in Mass Casualty Medicine *written by Griffin Trotter MD, PhD attempts to fill a timely void in this area by examining the ethics of coercion in times of public health disasters.

The seven chapters logically explain the dynamics of coercion, the ethical basis of public health, public health legitimacy, the role of public health "experts", public deliberation prior to these events, the role of leadership, and decisions for particular coercive actions. The central argument is the optimal balance between security and liberty. The author argues that in Mass Casualty Medicine, the Clinical paradigm is replaced by the Rescue Paradigm in which it is necessary to save lives and minimize aggregate morbidity. Clinicians in the trenches might find exception to the argument, as it may be impossible to easily switch from one paradigm to another.

Similar ethical and philosophical arguments throughout the book attempt to justify that public deliberation prior to mass casualty events, the use of the modus Vivendi approach (rather than rational consensus approach) which favors compromise rather than consensus, and decision-making at the local level are necessary in times of public health disasters. These arguments are somewhat logical and often supported by recent examples (eg decision-making during the events of September 11 to highlight tactical leadership failures). However, prior deliberation cannot prepare physicians to act in a certain way during disasters, as decisions are based on intuitive responses, which combine scientific evidence, experience and values.

Throughout the book, ethical principles such as 'utilitarianism' by John Rawls are explained, whereas others (eg "civic teleology") may be challenging for the novice for whom the bibliography may prove useful. However, repeated references to the works of a few authors (eg Lawrence Gostin and MSHEPA) limit the bibliography's overall educational value as a comprehensive resource.

Public health readers interested in a broader view of global public health ethics in disasters may be disappointed by the absence of a global perspective as the examples are limited to the United States. The use of coercion during the SARS epidemic in Canada and global public health disasters such as the HIV epidemic in Africa, the Iraq war and the use of coercion and torture by medical professionals are ignored. Using the controversial ticking time-bomb example, the author argues that, "in certain instances the refusal to use torture would be morally reprehensible "[pg 106].

The author criticizes the role of the media during hurricane Katrina for portraying New Orleans as a city in chaos. However he fails to note the powerful role of the media in showing the detrimental impact of the disaster and subsequent public health efforts on the marginalized. Predominantly poor African Americans living in the geographically marginalized areas without adequate means of transportation were affected both by the flood as well as by subsequent coercive attempts to shelter them in the Superdome.

*The Ethics of Coercion in Mass Casualty Medicine *promises much more than it delivers. The author's position on contemporary issues may not accurately reflect the prevailing diversity of opinion and surrounding controversy on these topics, and leaves the reader with more questions than answers. However, he deserves commendation for attempting to grapple with these challenging public health issues and *The Ethics of Coercion in Mass Casualty Medicine *may serve as starting point to stimulate much needed public debate.

## Competing interests

The author(s) declare that they have no competing interests.

## Acknowledgements

The author processing charge for this book review was supported in part by a grant from the Irwin Foundation.

